# BTG Interacts with Retinoblastoma to Control Cell Fate in *Dictyostelium*

**DOI:** 10.1371/journal.pone.0009676

**Published:** 2010-03-12

**Authors:** Daniele Conte, Harry K. MacWilliams, Adriano Ceccarelli

**Affiliations:** 1 Dipartimento Scienze Cliniche e Biologiche Università degli Studi di Torino, Orbassano, Italy; 2 Department of Biology II, Biozentrum, University of Munich, Munich, Germany; Centre for Genomic Regulation (CRG), Universitat Pompeu Fabra, Spain

## Abstract

**Background:**

In the genesis of many tissues, a phase of cell proliferation is followed by cell cycle exit and terminal differentiation. The latter two processes overlap: genes involved in the cessation of growth may also be important in triggering differentiation. Though conceptually distinct, they are often causally related and functional interactions between the cell cycle machinery and cell fate control networks are fundamental to coordinate growth and differentiation. A switch from proliferation to differentiation may also be important in the life cycle of single-celled organisms, and genes which arose as regulators of microbial differentiation may be conserved in higher organisms. Studies in microorganisms may thus contribute to understanding the molecular links between cell cycle machinery and the determination of cell fate choice networks.

**Methodology/Principal Findings:**

Here we show that in the amoebozoan *D. discoideum*, an ortholog of the metazoan antiproliferative gene *btg* controls cell fate, and that this function is dependent on the presence of a second tumor suppressor ortholog, the retinoblastoma-like gene product. Specifically, we find that btg-overexpressing cells preferentially adopt a stalk cell (and, more particularly, an Anterior-Like Cell) fate. No btg-dependent preference for ALC fate is observed in cells in which the retinoblastoma-like gene has been genetically inactivated. *Dictyostelium* btg is the only example of non-metazoan member of the BTG family characterized so far, suggesting that a genetic interaction between btg and Rb predated the divergence between dictyostelids and metazoa.

**Conclusions/Significance:**

While the requirement for retinoblastoma function for BTG antiproliferative activity in metazoans is known, an interaction of these genes in the control of cell fate has not been previously documented. Involvement of a single pathway in the control of mutually exclusive processes may have relevant implication in the evolution of multicellularity.

## Introduction

Among other genes involved in the control of proliferation and/or differentiation, is BTG2/PC3, originally identified as a regulator of neuronal cell differentiation, and subsequently found to be endowed with antiproliferative activity [1], [2]. *Btg* is considered a marker of neuronal birth in the development of rat cerebral cortex [3], and belongs to a family of genes whose members share the antiproliferative function as well as the conserved domain APRO, considered to be the signature of this gene family. In many cases *Btg* antiproliferative activity represses cyclin D1 and E transcription; here its effects depend on a functional retinoblastoma protein [4]. In other cases *Btg* acts via a retinoblastoma-independent pathway [4]. Notably, *Btg* is expressed during the last cell cycle preceding the neural progenitor's final choice of fate and may thus act while the cell cycle is still in progress. *Btg* could thus effect epigenetic reprogramming during the terminal S-phase.

An increasing number of reports have proposed a role for BTG2 as a coactivator-corepressor and/or an adaptor molecule modulating the activities of its interacting proteins. BTG has also been proposed to interact with and modulate the function of differentiation regulators such as Hoxb9 [5] and BMPx [6].

*D. discoideum* is an amoebozoan that feeds upon bacteria and proliferates indefinitely as long as a food source is present. Upon starvation cells cease dividing and undergo a complex series of differentiative and morphogenetic events leading up to a mature fruiting body in which 80% of the cells form spores which are suspended atop a thin cellular stalk. Spore and stalk precursors can be identified at earlier developmental stages where they sort out to form a spatial pattern, with prestalk cells in the front of the motile aggregate and prespore cells, amounting to 80% of the cell mass in the back. Strewn amongst the prespores are a few cells with morphological and biochemical characteristics resembling prestalk, these cells are named Anterior-Like Cells (ALC). During terminal differentiation ALC will form two disc shaped structures, the upper and lower cups, at the poles of the mature spore mass, as well as the basal disc at the bottom of the stalk [7].

Among the most interesting aspects of *Dictyostelium* development is a strong link between cell cycle and cell fate. Cells show preferences for the stalk or spore fate depending on their cell cycle position at the beginning of development (reviewed by [8]). When marked cells from synchronized cultures are mixed with an excess of cells from an asynchronous population and starved, S- and early G2-phase cells are preferentially found in the prestalk/stalk pathway, while cells in mid-late G2 preferentially form spores. Recent studies suggest that cell cycle position modulates the sensitivity of cells to the Differentiation Inducing Factor (DIF), a chlorinated hydroxyphenone made by cells of spore pathway that promotes stalk differentiation at later stages of development [9].

In an effort to elucidate the molecular basis of the link between cell cycle and cell fate we have recently characterized rblA, the *Dictyostelium* ortholog of retinoblastoma gene (Rb), and shown that it regulates cell fate preference; rblA expression is correlated with preference for prespore fate. It appears likely that the effect of cell cycle on differentiation pathway preference is mediated by rblA [10]. In the present work we describe *btg*, the ortholog of Btg2/PC3, and the first example of a non metazoan member of the A-PRO family. We also present evidence that it acts mainly as a regulator of ALC fate choice. Its action, as that of Btg2/PC3 in metazoans, is dependent on retinoblastoma gene function.

## Results

### Structure of the *D. discoideum btg* gene

**Figure 1:**
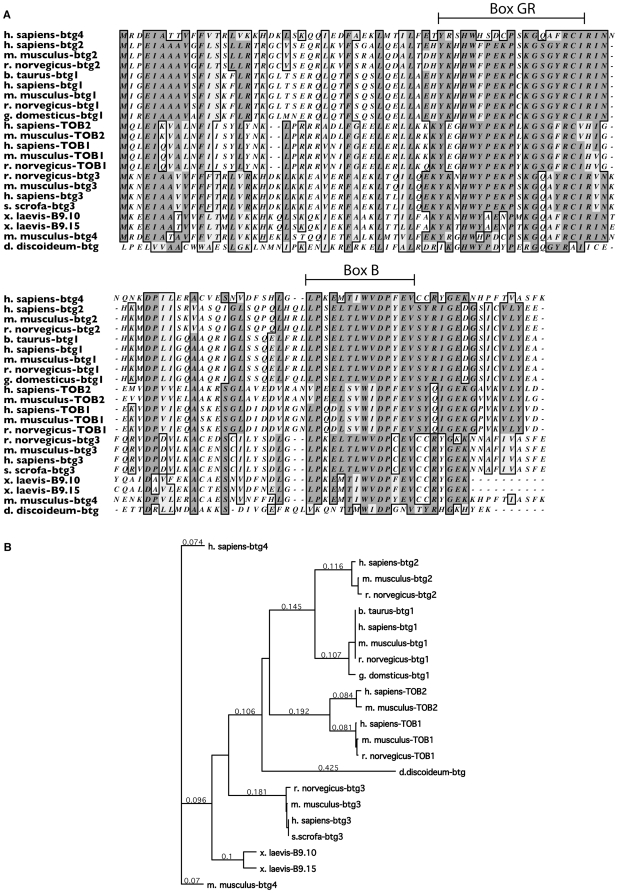
Alignment of *D. discoideum* BTG with other members of the A-PRO family and their phylogenetic relationship. a, The A-PRO domains of representatives members of the family have been aligned to *D. discoideum* BTG. Identical residues are in dark grey whereas conserved aminoacids are in light grey. GR (A) and B boxes are indicated with a line above the alignment. b, Phylogenetic relationship among Dd-btg and other APRO members. The alignment in (a) was used to generate the dendrogam.

*Btg* belongs to a family of genes sharing the A-PRO domain (pfam pf07742); these genes have roles as proliferation regulators as well as in the control of differentiation [3], [11]. We searched for the A-PRO domain in the *D. discoideum* genome, and found a single intronless gene predicting a protein of 423 amino acids (DDB_G0285069). The protein has 49% homology with the products of the *Pc3* and *Btg2* genes in mouse and rat respectively (Fig. 1). Within the A-PRO domain it is possible to identify box GR (also named boxA) and box B to which some of the known functions of this gene family have been mapped [11]. *D. discoideum* BTG showed a very high degree of conservation of the residues within box GR and box B, with 15/19 and 7/14 conserved or identical residues respectively. In its overall size *Dictyostelium* BTG protein resembled more closely the group of ToB members, while the match at the sequence level was better for the group of BGT2/PC3. In the dendrogram generated from the alignment BTG fell in a position almost equally distant from the two groups (Fig. 1). *D. discoideum* BTG also carries an additional N-terminal extension that is not observed in the other members of the family; its function is still unknown.

### Developmental expression of *btg*

**Figure 2:**
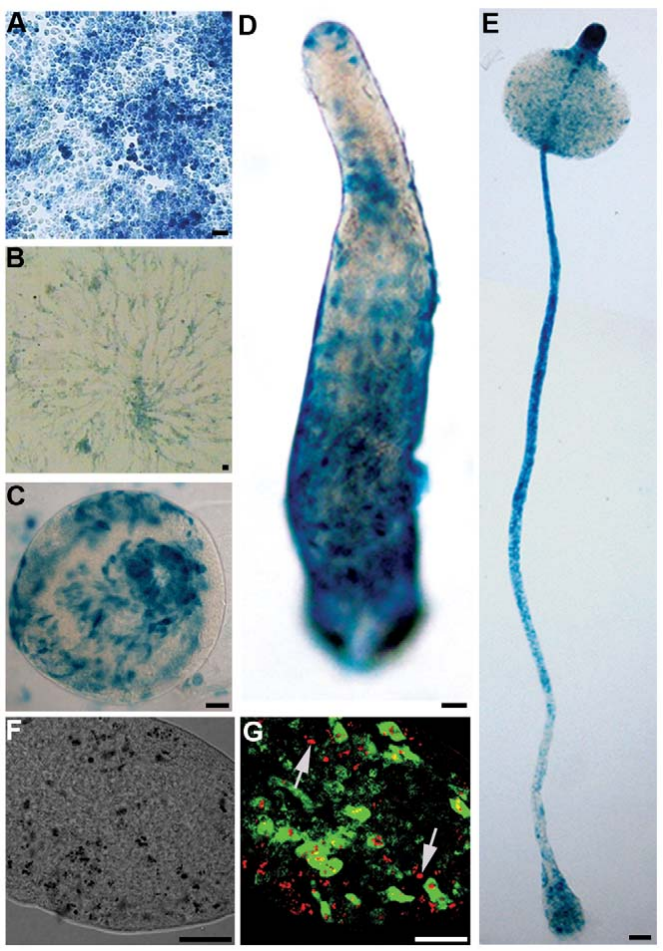
Transcription of *btg* during growth and development of *D. discoideum*. A–E, *D. discoideum* AX2 transformants expressing Pbtg-αGal at different stages. A, growing cells; B, aggregating cells; C, tipped mound; D, slug; E, culminant. Bars represent 20 µm. F–G, *Btg* is expressed in a subset of ALC cells. Cells expressing a labile GFP were stained with neutral red (NR) and allowed to develop to slug stage. F, brightfield showing NR in autophagic vacuoules (black spots); G, overlay of GFP (green) and NR (here in red). Arrows indicate cells only containing NR; bars represent 20 µm.

To analyse the expression of *btg* during *D. discoideum* life cycle we fused its regulatory region to the i-α-gal vector, encoding a labile version of the β-galactosidase (β-gal) [12]. Transformant *Dictyostelium* cells carrying the btg::αgal fusion showed vegetative as well as developmentally regulated reporter expression. During growth *btg* is heterogeneously expressed (Fig. 2A). A BrdU pulse chase labelling of transformant cells showed an effect of cell cycle position on *btg* expression, explaining the heterogeneity of expression seen in figure 2A (Fig. S1). In aggregating cells, *btg* was expressed in a hetereogeneous “salt and pepper” fashion (Fig. 2B). In late mounds, the btg-expressing cells were seen to spiral upward to the emerging tip, a pattern that is sometimes seen in cells destined for the prestalk zone (Fig. 2C) [13]. At the slug stage *btg* expression is found in scattered cells, mostly in the posterior half of the prespore territory, though sometimes it is also expressed in few cells scattered in the front half of the prespore compartment (Fig. 2D). Later, upper- and lower-cup specific as well as stalk-specific expression was observed (Fig. 2E). The continued activity of the short-lived reporter indicates that there is sustained gene expression throughout development and that the pattern observed at later stages does not merely depend upon sorting of cells expressing *btg* earlier on. We confirmed the reporter pattern by *in situ* hybridization (Fig. S2). In late development, the distribution of *btg* mRNA strongly resembled the pattern seen with the labile reporter, with the slight differences suggesting that the labile β-gal protein has a shorter half-life than the *btg* mRNA.

The position of the cells expressing *btg* at slug stage was compatible with Anterior-Like Cells (ALC) fate. ALC are a population of amoeboid cells dispersed among the prespores (psp) but expressing markers characteristic of the anterior prestalk (pst) portion of the slug, and are selectively stained with Neutral Red (NR) [7]. To determine whether *btg* expression is ALC specific we counterstained cells expressing the short-lived marker btg::ubi-GFP [14] with NR. The two markers colocalized in confocal sections thinner than a cell diameter (Fig. 2F, G), indicating that the GFP-expressing cells are ALC. It should be noted, however, that while all *btg* positive cells are ALC, not every ALC expresses *btg* (arrowed in Fig. 2G). *Btg* is thus expressed in a subpopulation of ALC.

### Btg is deregulated in DIF-signalling and rblAnull mutants

**Figure 3:**
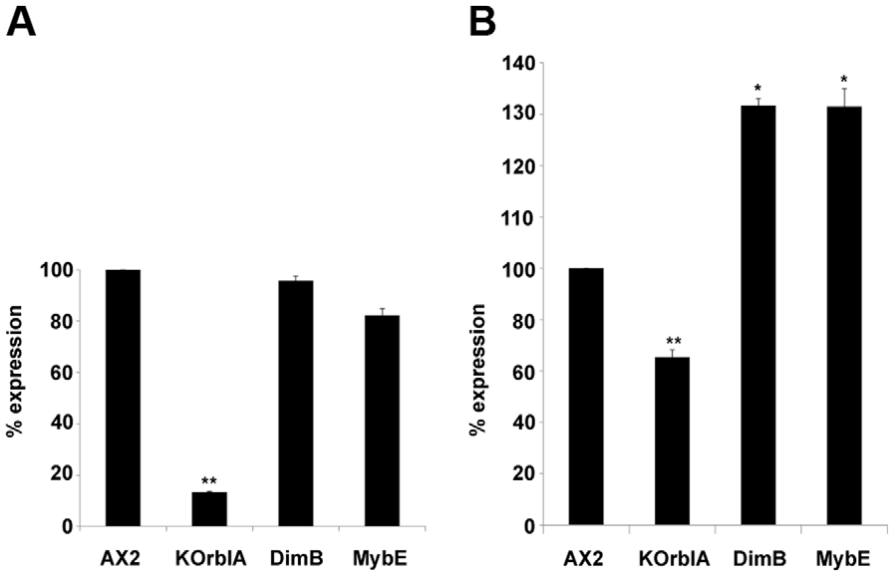
RB and DIF control *btg* expression during growth and development. Dicytostelium AX2, rblA-KO, dimB^−^ and mybE^−^ were transformed with Pbtg-αGal and assayed for β-gal expression during growth (A) and at slug stage (B). The values are averaged from 3 independent experiments. In panel A expression in mutant strains was normalized to expression in AX2 cells. In panel B the ratio of developmental expression over vegetative expression of each mutant strain is expressed as a percentage of the same value calulated for AX2. *: P≤0.05; **: P≤0.01; T test with N = 3. Error bars indicate s.e.m.

The ALC are known to be heterogeneous, including populations that are dependent or independent from the *Dictyostelium* stalk cell morphogen DIF [15], [16], [17]. We assessed the effects of DIF signalling on *btg* expression in strains in which *DimB* and *MybE*, effectors in DIF signalling pathways, have been inactivated by insertional mutagenesis [18], [19]. During vegetative growth, when DIF signalling is inactive, the wt Ax2 and the DIF unresponsive *dimB^−^* and *mybE^−^* mutants express *btg* at comparable levels (Fig. 3A). During development the DIF signalling system is turned on and *btg* is overinduced in the DIF unresponsive mutants (Fig. 3B). This suggests that *btg* is expressed in a class of ALC whose differentiation is inhibited by DIF. Additional evidence comes from studies of *btg* expression in an *rblA* disruptant, which shows enhanced DIF sensitivity [10]. Here, as predicted, *btg* expression decreases during development (Fig. 3B). To rule out the possibility of a lack of the ALC population as a whole in the rblA disruptant we stained with neutral red Pbtg-αGal slugs of both AX2 and rblA KO cells and observed that the total population of ALC in the *rblA* disruptant is not decreased (Fig. S3).

### Function of *btg* and its interaction with *rblA*

**Figure 4:**
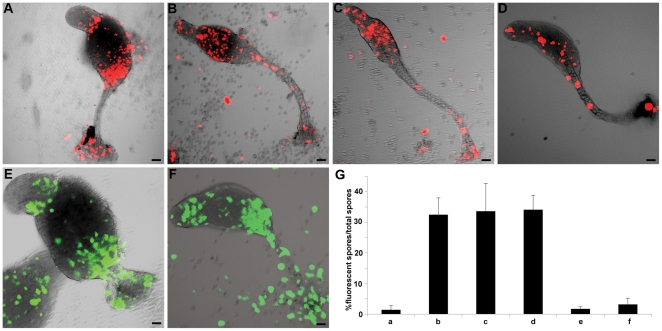
BTG controls cell fate and is RB-dependent. A–F, *Dictyostelium* cells constitutively expressing RFP or GFP and overexpressing btg were mixed at a 30∶70 ratio with unlabelled cells of the same strain and allowed to develop. In control experiments the corresponding RFP or GFP strains with no *btg* overexpression were used. A, RFP-btgOE AX2/AX2; B, RFP-AX2/AX2; C, RFP-btgOE rblA-KO/rblA-KO; D, RFP rblA-KO/rblA-KO; E, GFP btgOE rblA-KO/AX2; F, GFP rblA-KO/AX2; bars represent 20 µm; G, culminants from samples A–F were squashed on a microscope slide and the percentage of RFP- or GFP-positive spores was determinated. Error bars indicate s.e.m.

To study the role of BTG in growth and development of *D. discoideum* we placed a myc-tagged version of BTG under the strong constitutive actin15 promoter [20]. Cells overexpressing BTG showed little or no increase in doubling time (Fig. S4) indicating the absence of an antiproliferative effect. When the same cells were allowed to develop, morphology as well as developmental timing were overtly normal, but the overexpressors showed preference for the ALC fate. Thus, when we labelled *btg* overexpressing (btg-OE) AX2 cells (Fig. S4) with a constitutively expressed red fluorescent protein (RFP) [21], and mixed them with an excess of wild type cells, the labelled cells sorted to the upper and lower cups and outer basal disc, the three structures derived from the ALC of the slug (Fig. 4A).

**Figure 5:**
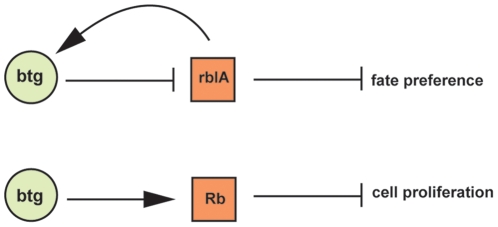
Interactions between BTG and RB in *D. discoideum* and mammalian cells. Comparison between the proposed interactions between BTG and RBLA regulating ALC fate in *D. discoideum* and the mammalian pathway. In the upper diagram BTG negatively controls RBLA, which in turn negatively controls ALC fate. In addition, RBLA depresses DIF sensitivity, leading to an indirect stimulation of BTG, this provides a feedback modulation of some, but perhaps not all BTG effects. In the lower diagram BTG acts positively on RB function, resulting in the antiproliferative effect observed in mammalian cells. In this case a feedback regulation of RB on BTG has not been described.

In mammalian cells the antiproliferative function of BTG is RB-dependent, and we have shown that RB function itself regulates fate choice in *D. discoideum* [10]. To understand whether BTG acts in an RB-dependent manner in the regulation of ALC fate, we repeated the mixing experiment in the *rblA* disruptant strain. BtgOE *rblA* disruptant cells labelled with A15RFP [22]were mixed at a 30∶70 ratio with *rblA* disruptant cells and allowed to develop. In this background no effect of *btg* overexpression was observed, as expected if also in *Dictyostelium* BTG function is dependent on RB (Fig. 4C). This observation places *btg* and *rblA* on the same functional pathway. We have previously demonstrated that *rblA* disruptant cells show a differentiation pathway preference at slug stage that is compatible with the ALC fate [10]. To determine whether the effect of BTG is entirely mediated by RB, we mixed *rblA*-disruptant cells labelled with constitutive GFP [23], with or without overexpression of btg, with an excess of wild type cells. The *rblA* disruptant cells showed the expected preference for the ALC fate, indicated by sorting to upper and lower cup in the chimera. However, overexpression of *btg* produced no apparent enhancement of this fate preference (Fig. 4E). To show that the genetic interaction between *btg* and *rblA* affects differentiation rather than merely modulating sorting, we measured the fraction of fluorescent spores in the various chimeras. This was normal when both strains were wild type or when both strains carried either btg-OE or *rblA* disruption. A drastic reduction in spore formation was observed when marked cells carrying either btg-OE, *rblA* disruption, or both, were mixed with wild type cells. As with the observations on sorting, we saw no significant enhancement in the double mutant (Fig. 4G). All of our observations can be explained by the model shown in Fig. 5.

## Discussion

We have isolated and characterized the *D. discoideum btg* gene. In vertebrates *btg* has an antiproliferative function and is also known to be involved in the regulation of differentiative events, such as neuronal birth. *Btg* antiproliferative function can be exerted through the inhibition of cyclin D1 at the transcriptional level. This in turn keeps the tumor suppressor Rb complexed with E2F, thereby inhibiting G1-S transition. Thus, in mammalian cells, *btg* antiproliferative function is Rb-dependent.

In *D. discoideum* the antiproliferative role of *btg* during vegetative growth is undetectable, and cells overexpressing *btg* showed no effects on growth rate or cell morphology. This result was anticipated by our previous observation that genetic disruption of *Dictyostelium* Rb function does not affect proliferation, presumably as a consequence of the lack of detectable G1 phase in growing *Dictyostelium* amoebae [10]. However while *rblA*, the *Dictyostelium* retinoblastoma ortholog, is expressed at a very low rate during vegetative growth, *btg* is expressed at a higher level and in a cell cycle regulated fashion. Therefore a function during growth could reasonably be posited but must be a very subtle one.

Our study of *btg* expression during *Dictyostelium* differentiation shows that it is distributed in a salt and pepper pattern during aggregation, to become ALC and stalk specific at later stages. ALC are a population of cells scattered among the prespore cells in the back of the slug with some of the features of the anterior pre-stalk cell in the tip. We found *btg* expressed in a subpopulation of ALC, consistent with the notion that ALC are a heterogeneous population based on the expression of pre-stalk specific markers [24]. Thus *btg* can be used as a marker for the identification of an ALC subpopulation and it will be interesting to study its overlap with presently known pre-stalk and ALC-specific markers.

We observe that *Dictyostelium* strains hypersensitive or unresponsive to DIF downregulate or upregulate respectively, a reporter construct driven by the *btg* promoter in ALC cells. The simplest explanation is that DIF negatively regulates *btg* expression. Direct evidence to support this notion would be provided by assaying the activity of DIF on the transcriptional activity of the *btg* promoter. However this experiment would not be easily interpretable due to the complex nature of the *btg* promoter, driving expression in ALCs as well as in stalk cells. The result of a monolayer DIF induction assay could be represented by the balance of two opposing activities of DIF: inhibition of ALC specific expression and induction of stalk-specific expression.

Other authors have found that components of the ALC population are directly induced by DIF and contribute to the formation of the basal disc and lower cup, ancillary stalk structures that form during morphogenesis [17]. This would suggest that the *btg*-positive cells that we observe are a different subpopulation of ALC.

However in the next set of experiments on *btg* function we show that its expression is one of the cues that predisposes undifferentiated amoebae to the ALC fate, and to the formation of the basal disc and upper and lower cups. The simplest explanation to this contradiction is that DIF signalling is not the only mechanism controlling the differentiation of the ALC forming basal disc and upper and lower cups.

We have shown that *btg* and *rblA* are on the same pathway controlling preference for ALC fate. *RblA^−^* phenotype shows preference for the ALC fate [10] suggesting that Rb function negatively regulates this fate. *Btg* overexpression phenocopies rblA^−^ fate preference but has no effect in a rblA^−^ background. Thus BTG controls preference for the ALC fate in an Rb-dependent fashion, and the interaction between BTG and Rb is conserved between amoebozoans and mammals, though with different effects. The formation of basal disc and upper and lower cup was used in our work as an indicator for ALC fate but also implied the ability of scattered ALC cells to sort to the final structures during culmination. The presence of btg-overexpressing *rblA^−^* spores shows that *rblA* mediates *btg* control directly on fate choice rather than being involved in secondary aspects such as cell motility.

In mammalian cells, BTG suppresses RB phosphorylation and upregulates RB function. In *Dictyostelium*, *btg* overexpression phenocopies *rblA* disruption, so that the relationship appears reversed. We see two ways in which these seemingly contradictory observations could be reconciled. One is to suppose that the relevant target genes depend, not on unphosphorylated (active) RB, but on phosphorylated, or perhaps hemiphosphorylated RB; this form would be lacking in both the btg-OE and the *rblA* disruptant. Another possibility is that the genes necessary for cell type choice depend on N-terminal determinants of the *Dictyostelium* RB protein. In the *rblA* disruptant the conserved Rb-A and Rb-B sequences are replaced by a resistance cassette, but upstream sequences are intact so that an N-terminal protein fragment may be present. Either one of these possibilities would be interesting, as most known effects of mammalian RB on the control of cell cycle progression are dependent on the unphosphorylated form, and relatively little is known about the function of the conserved RB N-terminus.

We have shown that the metazoan tumor suppressors BTG and RB collaborate to control cell differentiation in the amoebozoan *Dictyostelium*. The same molecules interact in mammalian cells to control cell cycle progression, thus it appears that the same pathway can regulate mutually exclusive processes. It is possible that cell cycle control function arose primarily in the ancestor of amoebozoa and metazoa, to be recruited to the control of cell differentiation at later times when multicellularity evolved. However, *Dictyostelium* and metazoan development as well as multicellularity differ substantially, and it will be necessary to study the role of btg-Rb interactions in metazoan differentiation. At the same time, involvement of the btg-Rb functional link in the control of cell cycle in metazoans impinges on the possibility to identify concomitant specific roles in the control of cell differentiation. Our work in *Dictyostelium* opens new avenues of investigation for BTG and potentially other tumor suppressors.

## Materials and Methods

### *Dictyostelium* strains and basic methods

Axenic strain AX2 [25] was used in all experiments. Cells were transformed by electroporation [26] and selected with the appropriate antibiotic at 10–20 µg/ml. Cell growth and developmental conditions [27], BrdU labelling and β-gal histochemical and colorimetric assays [12] were as previously described.

### Molecular biology

All enzymatic reactions were performed as recommended by the manufacturers. The *btg* promoter was defined as the region from −923 to +3relative to the AUG cloned in i-α-gal plasmid [12] yelding Pbtg-αgal. In the overexpression construct, btg-OE, the *btg* coding sequence, preceded by a *myc*-epitope, was driven by the actin15 promoter in a pDD17 backbone [28]. For *in situ* hybridization, riboprobes were synthesized corresponding to the entire *btg* coding sequence and specimens were prepared and hybridized as previously described [29], except for Protease K (Sigma-Aldrich) used at 10 ug/ml for 10′, hybridisation temperature at 48°C, and final detection performed using FastRed TR-Naphthol (Sigma-Aldrich) according to manufacturer instructions. Sequence alignment and dendrogram generation were performed with the PHYLIPS software package [30].

## Supporting Information

Figure S1 Cell cycle regulated expression of *btg*. Pbtg-αGal transformants labelled with BrdU for 30 min were harvested at 1 hour intervals, fixed and stained for BrdU and β-gal. The frequency of double positive cells over BrdU positive cells was determined by counting several fields and then plotted over time. T tests (n = 4) are indicated by asterisks: *  =  P<0,05; **  =  P<0,01. Data are presented as mean and s.d of three independent experiments.
(0.39 MB TIF)

Figure S2 The 923 bp upstream of the *btg* AUG are sufficient to confer quantitatively correct and specific expression. a, pattern of expression of construct; b, pattern of expression of *btg* mRNA detected by *in situ* hybridisation. The white dotted line represents the shape of the aggregate. Bars represent 20 µm. There are small differences between β-gal and *in situ* hybridization patterns at the entrance of the stalk tube that could be explained assuming differential half-lives of β-gal protein and *btg* mRNA. The enzymatic assay allows very little amounts of activity to be detected, while a longer time is necessary for the mRNA to accumulate to a level detectable in the *in situ* hybridization. During this time the stalk is continuously elongated and the cells formerly at the entrance are found further down along the stalk.
(1.52 MB TIF)

Figure S3 *btg* expression is specifically downregulated in rblA disruptants. Cells of the AX2 (wt) and rblA disruptant strains carrying vector Pbtg-αGal were stained for β-gal activity, showing complete lack of *btg* expression in the rblA disruptant (A and C). To rule out the possibility that this pattern was the consequence of the loss of the ALC population as a whole in the rblA mutant, in the same experiment cells were vitally stained with neutral red, allowed to develop to slug stage, and observed. Expression of *btg* is downregulated in the rblA disruptant slug but the total amount of ALC is comparable to AX2 (B and D).
(2.40 MB TIF)

Figure S4 Overexpression of *btg* in wild type and rblA and its effects on cell growth. Proteins from AX2 and rblA disruptant slugs transformed with A15mycbtg were separated by SDS-PAGE and an anti-cmyc antibody (9E11 - Sigma-Aldrich) was used to detect the tagged BTG. A: autoradiography of the western blot probed with anti-cmyc and detected with ECL. An anti-actin antibody was used to normalise for protein content. B: quantitation of the image in (A) after the normalization. Densitometry was performed by analysing the scanned autoradiography with ImageJ software. C: Overexpression of *btg* does not affect growth rate. Growth of *Dicyostelium* AX2 cells untransformed or transformed as in (A) was monitored at indicated time intervals. Open squares: btgOE; open triangles: untransformed AX2 cells.
(2.21 MB TIF)
