# Supplementary figures and images for: BTG Interacts with Retinoblastoma to Control Cell Fate in Dictyostelium
Source: PLoS One. 2010 Mar 12;5(3):e9676. doi: 10.1371/journal.pone.0009676 (PMC2837350; doi:10.1371/journal.pone.0009676)

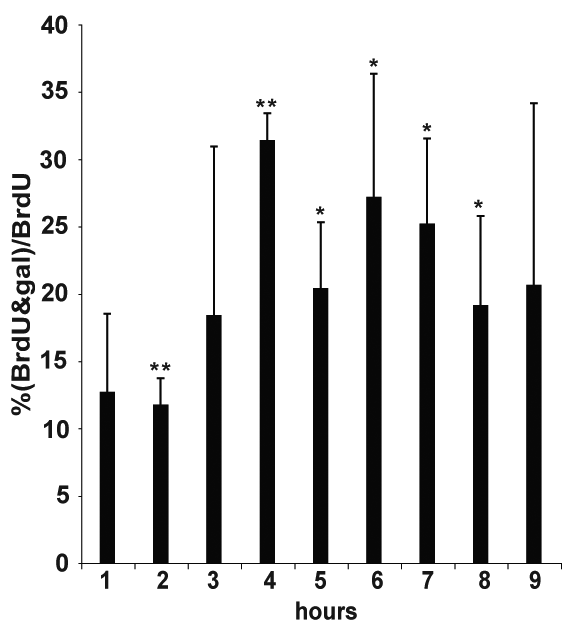

Supplement: Figure S1 — Cell cycle regulated expression of btg. Pbtg-αGal transformants labelled with BrdU for 30 min were harvested at 1 hour intervals, fixed and stained for BrdU and β-gal. The frequency of double positive cells over BrdU positive cells was determined by counting several fields and then plotted over time. T tests (n = 4) are indicated by asterisks: * = P<0,05; ** = P<0,01. Data are presented as mean and s.d of three independent experiments. (0.39 MB TIF) [file pone.0009676.s001.tif]

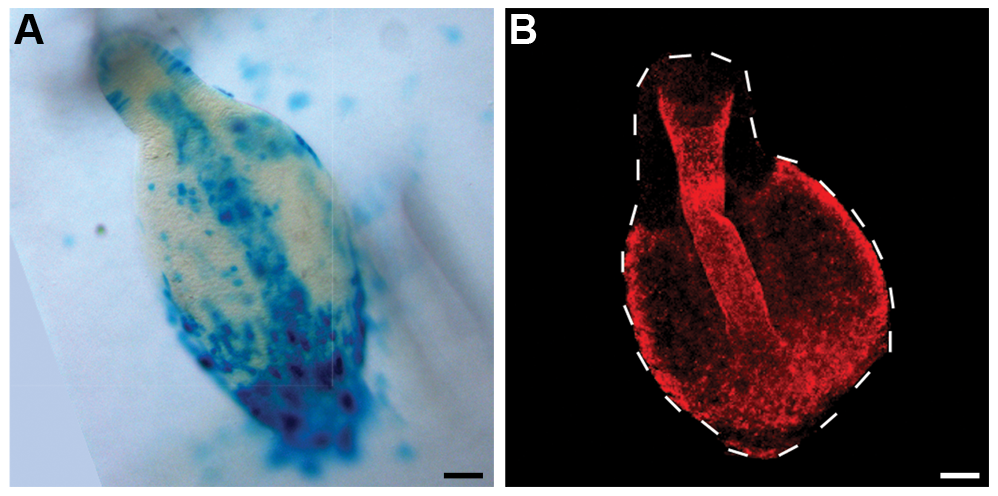

Supplement: Figure S2 — The 923 bp upstream of the btg AUG are sufficient to confer quantitatively correct and specific expression. a, pattern of expression of construct; b, pattern of expression of btg mRNA detected by in situ hybridisation. The white dotted line represents the shape of the aggregate. Bars represent 20 µm. There are small differences between β-gal and in situ hybridization patterns at the entrance of the stalk tube that could be explained assuming differential half-lives of β-gal protein and btg mRNA. The enzymatic assay allows very little amounts of activity to be detected, while a longer time is necessary for the mRNA to accumulate to a level detectable in the in situ hybridization. During this time the stalk is continuously elongated and the cells formerly at the entrance are found further down along the stalk. (1.52 MB TIF) [file pone.0009676.s002.tif]

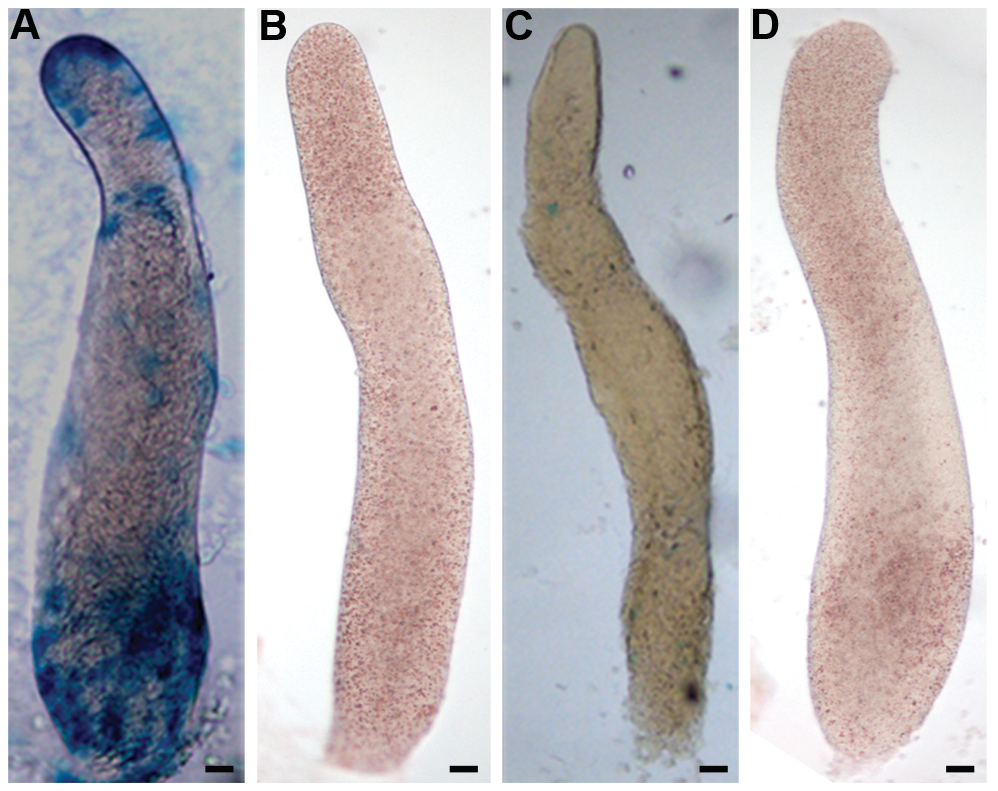

Supplement: Figure S3 — btg expression is specifically downregulated in rblA disruptants. Cells of the AX2 (wt) and rblA disruptant strains carrying vector Pbtg-αGal were stained for β-gal activity, showing complete lack of btg expression in the rblA disruptant (A and C). To rule out the possibility that this pattern was the consequence of the loss of the ALC population as a whole in the rblA mutant, in the same experiment cells were vitally stained with neutral red, allowed to develop to slug stage, and observed. Expression of btg is downregulated in the rblA disruptant slug but the total amount of ALC is comparable to AX2 (B and D). (2.40 MB TIF) [file pone.0009676.s003.tif]

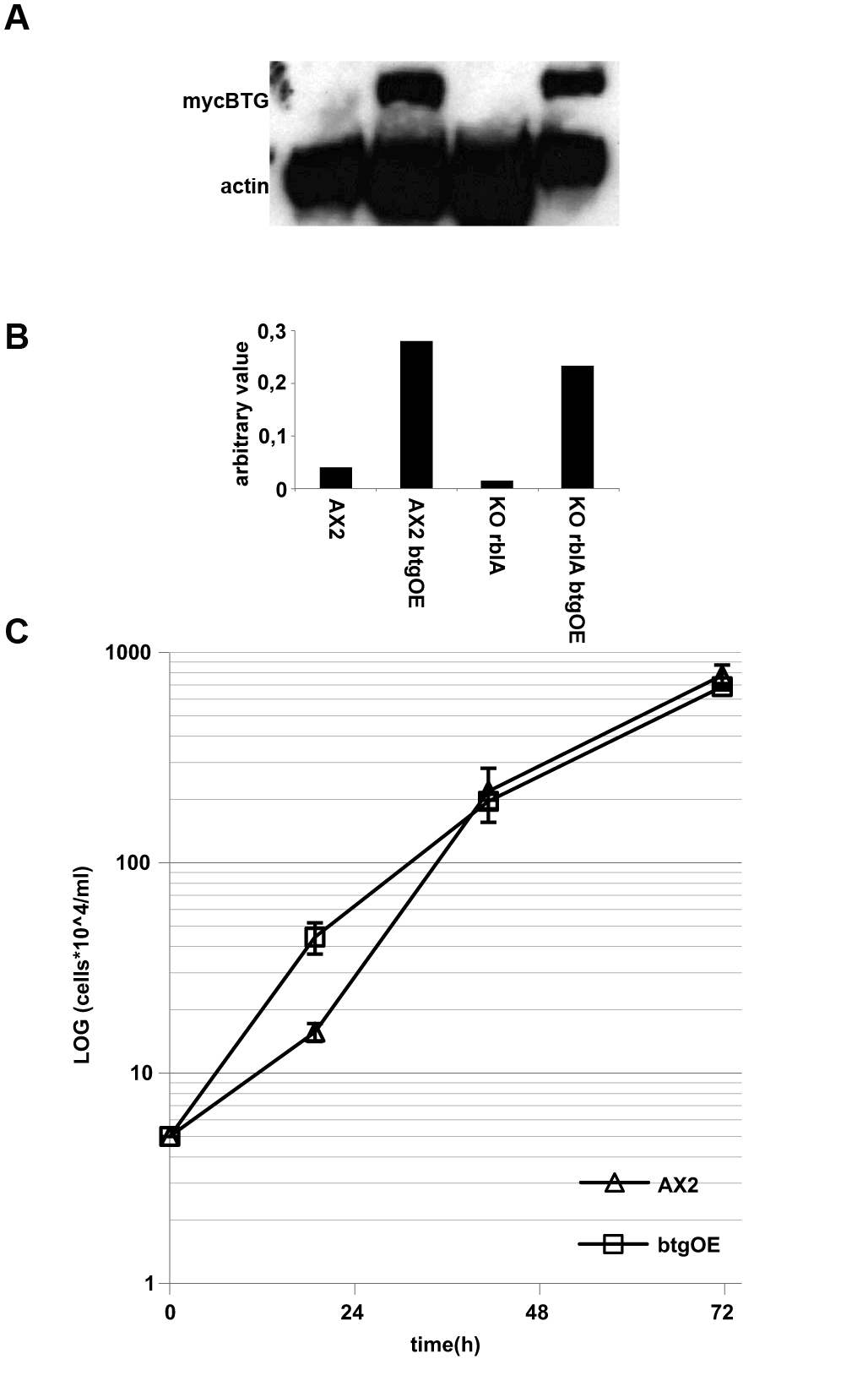

Supplement: Figure S4 — Overexpression of btg in wild type and rblA and its effects on cell growth. Proteins from AX2 and rblA disruptant slugs transformed with A15mycbtg were separated by SDS-PAGE and an anti-cmyc antibody (9E11 - Sigma-Aldrich) was used to detect the tagged BTG. A: autoradiography of the western blot probed with anti-cmyc and detected with ECL. An anti-actin antibody was used to normalise for protein content. B: quantitation of the image in (A) after the normalization. Densitometry was performed by analysing the scanned autoradiography with ImageJ software. C: Overexpression of btg does not affect growth rate. Growth of Dicyostelium AX2 cells untransformed or transformed as in (A) was monitored at indicated time intervals. Open squares: btgOE; open triangles: untransformed AX2 cells. (2.21 MB TIF) [file pone.0009676.s004.tif]
